# The effect of pristine carbon-based nanomaterial on the growth of green gram sprouts and pH of water

**DOI:** 10.1186/1556-276X-9-583

**Published:** 2014-10-21

**Authors:** Xiaolin Li, Zhihua Zhou, Dejiong Lu, Xinwei Dong, Minghan Xu, Liangming Wei, Yafei Zhang

**Affiliations:** 1 Key Laboratory for Thin Film and Microfabrication Technology of the Ministry of Education School of Electronics, Information and Electrical Engineering Shanghai Jiao Tong University, Shanghai 200240, China; 2 State Key Laboratory of Electronic Thin Film and Integrated Devices, School of Microelectronics and Solid-state Electronics, University of Electronic Science and Technology of China, Chengdu 610054, China

**Keywords:** Toxicity, Bean sprout, Carbon-based nanomaterials

## Abstract

We examined the toxicity of four carbon-based nanomaterials (unmodified) by using carbon quantum dots (CQDs), graphene quantum dots (GQDs), graphene oxide (GO), and single-walled carbon nanotubes (SWCNTs) to cultivate bean sprout. Results showed that the toxicity of these four carbon nanomaterials increases with the increasing of concentration and cultivating time. In addition, pH test was applied to study the effect of carbon-based nanomaterials on water. pH of culture solution displayed unconspicuous dose-dependent, but nanomaterials indeed have a considerable impact on the pH even at low concentration.

## Background

Nanotechnogy has undergone rapid development in recent decades. But at the same time, it has aroused a vast concern on the safety of nanomaterials [1-5], especially in biomedicine [6,7] and food science [8,9], and its effect on the environment (air, water, and soil) [10]. So far, there is not a universally accepted standard to test and assess the toxicity of nanomaterials in their practical applications. In effect, risk assessment of nanomaterials is a challenge, which needs to consider its size distribution, morphology, structure, solubility, surface charge, mass concentration, and uncertainties in practice, but indeed essential [11,12]. A large research on testing the toxicity of nanomaterials had been implemented to make sure that it could be used *in vivo* and *in vitro* as nanocarrier or bioimaging [13,14], and generally, the dosage used to examine is principally only in microgramme [6,7,15-17]. However, in fact, the dosage released to the environment or contained in a product is more than that tested in most researches. Thus, it is vital to test the toxicity of nanomaterials in a wide range of mass concentration.

Among multifarious nanomaterials, carbon-based nanomaterials have inspired intensive research interests due to their unique physical and chemical properties. Various forms of carbon-based nanomaterials have been actively explored with novel applications such as in material science [16-19], biosensing [20-22], biomedicine [7,15-17], and nanoelectronics [23,24]. Recently, the toxicity of CQDs was tested via cultivating bean sprouts and cells in culture solution which contains nanomaterials [5,25]. Except for using the cell to examine the toxicity of nanomaterials, bean sprouts are also a reasonable option because they are suitable for hydroponics and they are sensitive to toxins.They can also reflect the toxicity at the early stage of exposure.

In this study, we investigated the toxicity of four types of soluble pristine carbon-based nanomaterial (unmodified), carbon quantum dots (CQDs), graphene quantum dots (GQDs), graphene oxide (GO), and single-walled carbon nanotubes SWCNTs, through cultivating bean sprouts in a culture solution where the concentration of carbon-based nanomaterial ranged from 50 μg/mL to 1.2 mg/mL. To our knowledge, no systematic research has been made on their relative toxicity in such a large range of concentration. The length of green gram was measured as a parameter to judge toxicity, and then the distribution of CQDs in green gram was examined by direct visualization. Furthermore, pH test was applied to study the effect of carbon-based nanomaterials on water.

## Methods

### Materials

All reagents were analytical grade and were used without further purification. Citric acid was analytical-reagent grade and purchased from Aladdin Chemistry Co. Ltd (Aladdin Chemistry Co. Ltd, Shanghai, China). Graphite rod was purchased from Carbon Co., Ltd (Carbon Co., Ltd, Shanghai, China). Urea, methanol, dichloromethane, sodium hydroxide (NaOH), sodium nitrate (NaNO_3_), potassium permanganate (KMnO_4_), hydrogen peroxide (H_2_O_2_), and hydrochloric acid (HCl) were purchased from Sinopharm Chemical Reagent Co., Ltd (Sinopharm Chemical Reagent Co., Ltd, Shanghai, China). Green grams were obtained from the supermarket. Deionized water with a resistivity of 18.1 MΩ•cm was used for all experiments.

### Characterization

The morphologies of the samples were observed by transmission electron microscope (TEM, JEM-2100, JEOL, Akishima-shi, Japan), atomic force microscopy (AFM, Multimode Nanoscope, Digital Instruments, Santa Barbara, USA) and field emission scanning electron microscopy (FESEM, Carl Zeiss Ultra 55, Jena, Germany). The photoluminescent (PL) spectra were performed through a fluorescent spectrophotometer (F-4600, Hitachi, Japan). The ultraviolet-visible near-infrared (UV-vis-NIR) absorption spectrum was recorded by a UV-Vis-NIR spectrophotometer (Perkin-Elmer Lambda 950, Waltham, MA, USA).

### Preparation of carbon quantum dots

The modified method was based on previous report [25]. To a vigorously stirred 35 mL of deionized water, we added 10 g of citric acid and 10 g of urea. After the mixture turned to transparent solution, it was heated in a domestic microwave oven (800 W) for 5 min. Over the course, the solution was changed into black solid, which indicates the formation of CQDs. Then, the obtained solution was concentrated before transferring to a silica gel column. Next, the solution was eluted with mixture of methanol and dichloromethane in a ratio from 1:2 to 1:1 (*V*:*V*) to obtain N-doped CDs. Finally the resulting N-doped CQDs were evaporated into solid powders for further characterization and usage.

### Preparation of graphene quantum dots

Electrochemical method was applied to prepare GQDs [26]. Electrolysis process was performed on APS3005S. Graphite rod and carbon fiber were used as cathode and anode, respectively, and parallelly inserted into 100 ml of 0.1 M NaOH aqueous solution. After electrolysis overnight, the colorless solution turned to brown. And then, the resulting solution from the electrolysis was adjusted to neutral through sodium cation exchange resin. Further, it was purified by vacuum suction filtration to remove residue in solution, and GQDs powders were obtained by evaporation.

### Preparation of graphene oxide

Modified Hummer's method [27] was used to prepare GO. Briefly, 1 g of graphite was added to 25 ml of concentrated sulfuric acid solution with vigorous stirring at room temperature, and then NaNO_3_ (1.25 g) was added to the mixture. After continuous stirring for 1 h, the mixture was cooled to 0°C through ice-water bath. Next, 3.7 g of KMnO_4_ was slowly added in 2 h, after which the temperature was increased to 35°C, and the suspension was allowed to react for another 2 h. Afterward, 0.1 L of ice water and 3.5 mL of H_2_O_2_ were added to quench the oxidizing reaction. The prepared GO was filtered and washed with distilled water till the solution was neutral. Finally, GO got dried in an oven.

### Preparation of single-walled carbon nanotubes

The synthesis of SWCNTs was performed by arc discharge method as described earlier [28]. For further purification [29], 3 g of SWCNTs was transferred to muffle at 380°C for 2 h. Under sonication, the oxidized SWCNTs were added to 300 ml of concentrated hydrochloric acid, and then the suspension was centrifuged and washed with deionized water till neutralized. The purified SWCNTs were obtained after drying in an oven at 150°C for 24 h.

### Cultivation of the bean sprouts with different concentration of carbon-based nanomaterial

For the experimental group, 6 mL of different concentration (50, 100, 400, 800, and 1.2 mg/mL) of carbon-based nanomaterial aqueous solution was injected to every culture dish with 5.5 cm in diameter. Every experiment consists of four parallel tests, and the weight deviation of 15 green grams in every dish was 0.03 ± 0.02 g among each group. Under room temperature (about 25°C to 27°C), the dishes were placed, as far as possible away from light, without lids so as to make sure the sufficient oxygen supply. During the growth, appropriate water was supplemented to the dishes to keep the fixed volume of solution due to the evaporation. The average length (*n* = 60) of bean sprouts was measured every 24 h within 120 h, and their growth situation was photographed. For the control group, it is only changing carbon-based nanomaterial solution to deionized water which was the solvent for carbon-based nanomaterial in this study.

## Results and discussion

**Figure 1:**
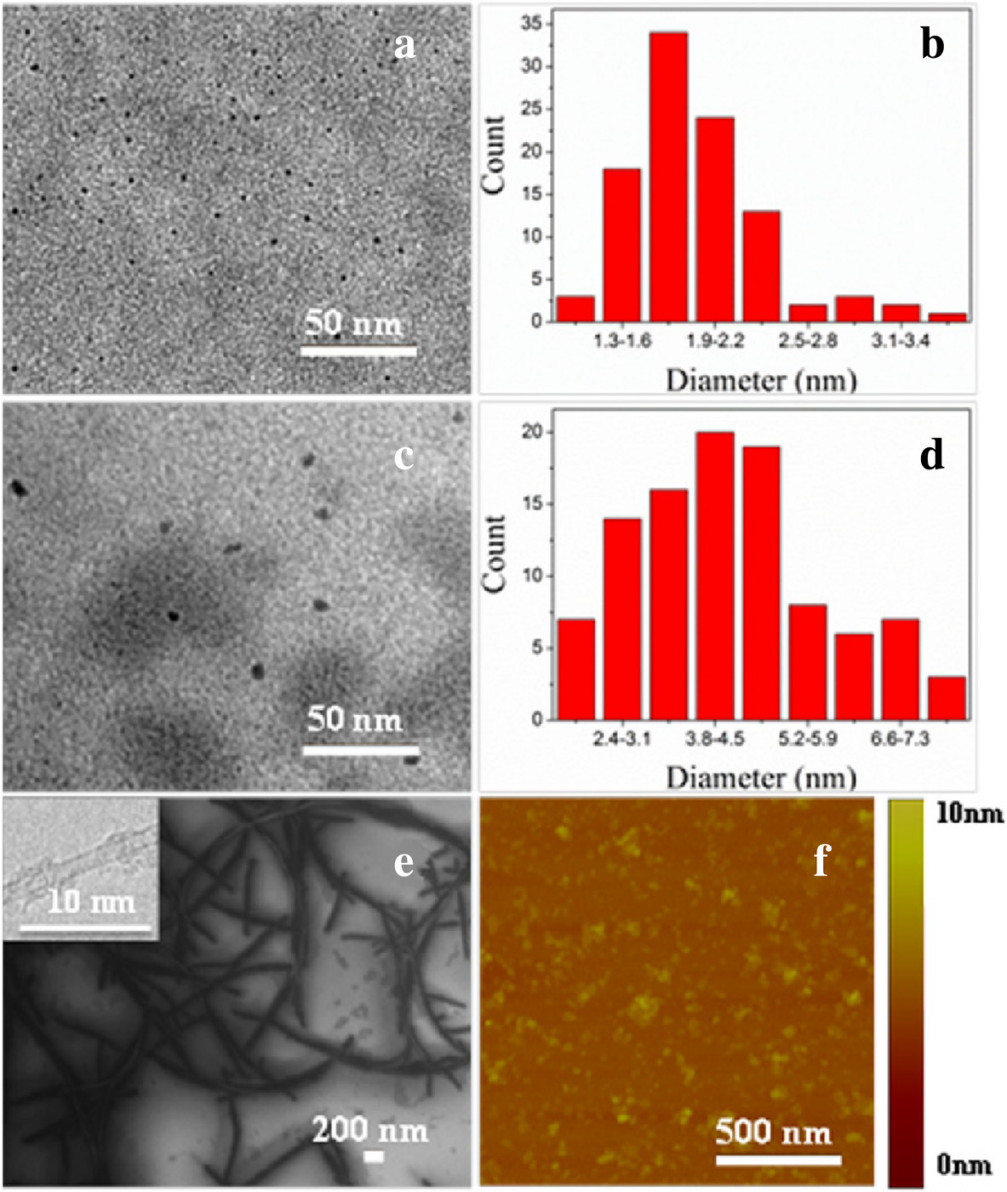
**TEM, SEM, and AFM images and particle size distribution.** TEM images of **(a)** CQDs and **(c)** GQDs. Particle size distribution of **(b)** CQDs and **(d)** GQDs. SEM image of **(e)** SWCNTs, and inset is the TEM image. AFM images of **(f)** GO.

**Figure 2:**
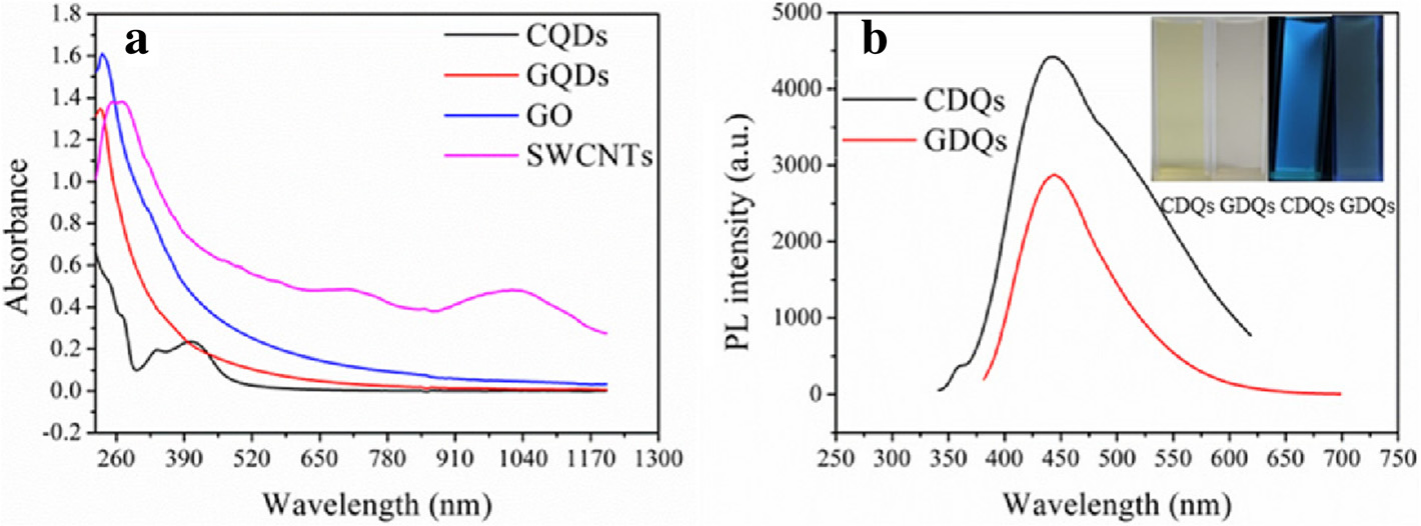
**UV-vis-NIR absorption and PL spectra. (a)** UV-vis**-**NIR absorption spectrum of four carbon-based nanomaterials. **(b)** PL spectrum of CQDs and GQDs excitated by 360 and 320 nm, respectively. Inset are photos of CQDs and GQDs solution under sunlight (the left two) and under dark (the right two) irradiated by a 365-nm UV beam.

Figure 1 shows the typical images of nanoparticles and particle size distribution by counting the average sizes of 100 nanoparticles (Figure 1b,d). CQDs and GQDs are uniform in size and present a nearly spherical shape with an average size of 2.10 and 5.00 nm, respectively (Figure 1a,c). GO and SWCNTs with a small size are obtained by ultrasonic cell disintegrator, and then the suspension was filtered with 0.22 μm cellulose filtration membrane to remove large and agglomerated particles (Figure 1e,f). UV-vis-IR absorption spectrum displays obvious characteristic absorption peak of four carbon-based nanomaterials (Figure 2a). The absorption peaks of GQDs and GO both center at approximately 230 nm, and SWCNTs exhibit two broad peaks around 700 and 1,050 nm. CQDs have three absorption peaks at 270, 340, and 405 nm. The PL spectrum of CQDs and GQDs demonstrate emission peak at 445 and 438 nm, respectively (Figure 2b). All these imply the successful preparation of the nanomaterials.

**Figure 3:**
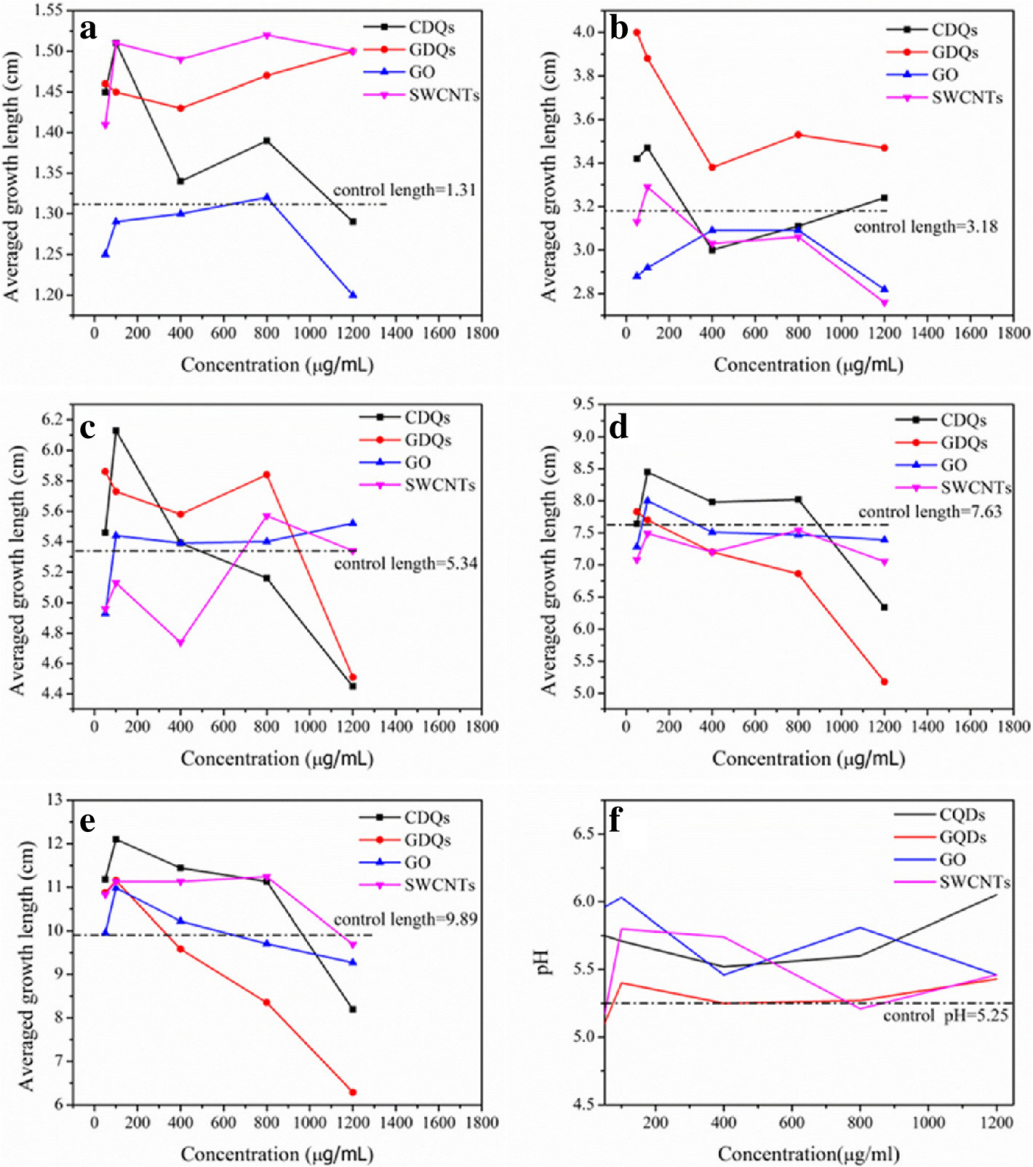
**Average growth length (*n *= 60) of bean sprouts with time in different culture media. (a)** 24, **(b)** 48, **(c)** 72, **(d)** 96, and **(e)** 120 h. **(f)** The pH curves of culture solution with different concentration after growing bean sprouts in 120 h.

**Figure 4:**
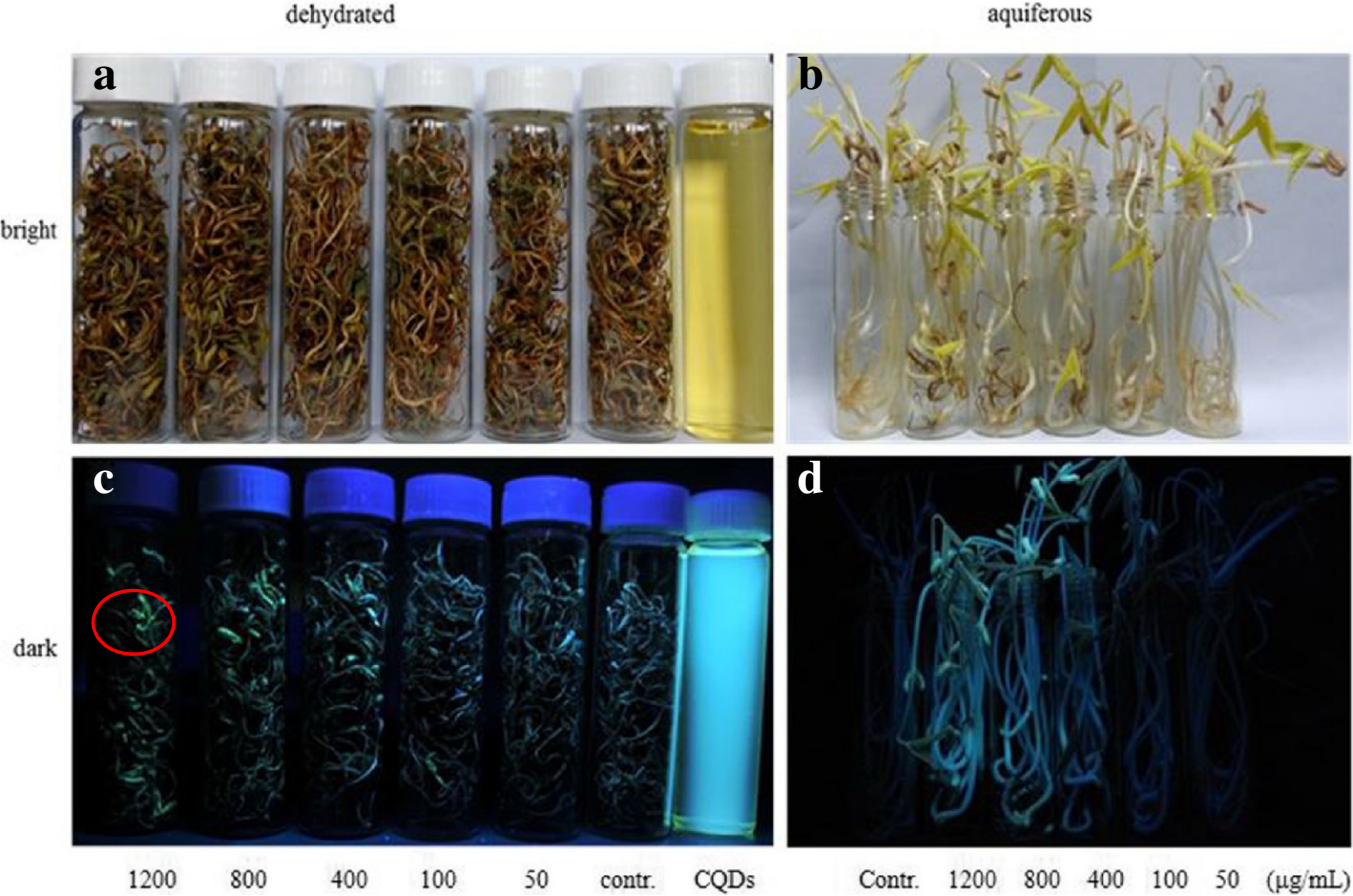
**Images of dehydrated and aquiferous bean sprouts.** Images of **(a)** dehydrated bean sprouts and **(b)** aquiferous bean sprouts in bright. Images of **(c)** dehydrated bean sprouts and **(d)** aquiferous bean sprouts in dark irradiated by a 365-nm UV beam. The solution in **(a)** and **(c)** is CQDs.

All of these nanomaterials, which consist of carbon element but with different structure, exhibit different effect on the length of the bean sprouts. Additional file 1 (Figure S1-S4) shows some typical growth situations of the bean sprouts with time. In the initial stage (before 72 h), no obvious difference is observed among all the samples; however, nanoparticles with high concentration (≥800 μg/mL) show inhibitive effect on this plant after 96 h (Figure 3a,b,c,d,e). Figure 3e shows the detailed comparison of the averaged growth lengths (*n* = 60) in all groups, the length of bean sprouts generally decreases with increasing the concentration of nanomaterials, and it is clear to see that GQDs exhibit remarkable inhibition at high concentration and more dose-dependent than others. Except for GQDs, the other three can promote the growth of bean sprout at the concentration less than 800 μg/mL, demonstrating nontoxicity of these nanomaterials. But for the high concentration (1,200 μg/mL), all these nanoparticles show different degree of inhibition. It is worth noting that the length curve of SWCNTs changes more obviously over the 96 h due to its low solubility (compared to the other three carbon-based nanomaterials), and the final results (Figure 3e) imply that SWCNTs display less dose-dependent and toxicity after a complete growth circle (120 h). Interestingly, it finds that 100 μg/mL seems to be the most conducive concentration for the growth of green gram sprouts. All these imply that the toxicity of these nanoparticles is time/dose-dependent. So dosage should be concerned in practice, especially particle with such small size can permeate throughout the cytoderm and cytomembrane with good biocompatibility [25,30,31]. As shown in Figure 4, bean sprouts cultivated at high concentration of CQDs emit green fluorescence as marked by red circle when irradiated by a 365 nm UV beam in dark (Figure 4d); however, no fluorescence was observed at low concentration. After drying at room temperature, bean sprouts still possess fluorescence (Figure 4c).In addition, the impact of nanomaterials on environment also deserves our full attention. Although the initial concentration of nanoparticles presents irregular influence on pH mainly due to dispersibility of nanoparticles in water, the pH indeed changes with different degrees compared to control group over all the concentration (Figure 3f), which would affect aquatic organism and even lead them to toxicosis.

## Conclusions

Through cultivating bean sprouts, the biomedical and environmental safety of four widely used pristine carbon-based nanomaterials have been systematically investigated. The growth of bean sprouts shows dose-dependent on the concentration of carbon-based nanomaterials, for which 100 and 1,200 μg/mL exhibit obvious promotion and inhibition, respectively. Moreover, nanomaterials indeed have a considerable impact on the pH of water even at low concentration. Thus, when utilizing the excellent properties of a nanomaterial, we should also consider its negative effect, especially to the environment and find out the related solutions. Risk assessment of nanomaterials is a long-term and complicated mission, and we believe that these results would provide valuable information for nanomaterials in practical applications.

## Competing interests

The authors declare that they have no competing interests.

## Authors' contributions

LXL performed most of the experiment and wrote the manuscript. ZHZ helped analyze the characterization results. DJL cultivated the bean sprout. XWD characterized the four carbon-based nanomaterials. MHX prepared the carbon quantum dots. LMW and YFZ supervised all of the study and provided financial support. All authors read and approved the final manuscript.

## Authors' information

Xiaolin Li is a PhD candidate at Prof. Yafei Zhang's group in Shanghai Jiaotong University. Her research focus now includes the preparation of gold nanoparticles and the application of carbon-based nanomaterials in biomedicine.

Zhihua Zhou is a lecturer in State Key Laboratory of Electronic Thin Film and Integrated Devices, University of Electronic Science and Technology of China, Chengdu, China. His research focus now includes the preparation of semiconductors and their device applications.

Dejiong Lu is a MS candidate at Prof. Zhang's group in Shanghai Jiaotong University. His research focus now includes nanomaterials and their application for sensor and solar cells.

Xinwei Dong is a PhD candidate at Prof. Yafei Zhang's group in Shanghai Jiao Tong University. His research focus now includes the preparation of carbon quantum dots and carbon-based nanomaterilas.

Minghan Xu is a PhD candidate at Prof. Zhang's group in Shanghai Jiaotong University. His research focus now includes the preparation of carbon quantum dots and their application in bioimaging.

Liangming Wei is currently a professor at Shanghai Jiaotong University, China. His research interests include preparation of carbon-based nanomaterilas and their use for in sensors and energy storage.

Yafei Zhang is currently a professor at Shanghai Jiaotong University, China. His research interests include synthesis of gas-sensing nanomaterials and nanomaterials and their applications in nanodevice.

## Supplementary Material

Additional file 1: Figure S1-S4 Photograph of the bean sprouts. Typical growth situations of the bean sprouts with time. Click here for file
