# Supplementary figures and images for: The effect of pristine carbon-based nanomaterial on the growth of green gram sprouts and pH of water
Source: Nanoscale Res Lett. 2014 Oct 21;9(1):583. doi: 10.1186/1556-276X-9-583 (PMC4207633; doi:10.1186/1556-276X-9-583)

**Additional file 1: Figure S1-S4**


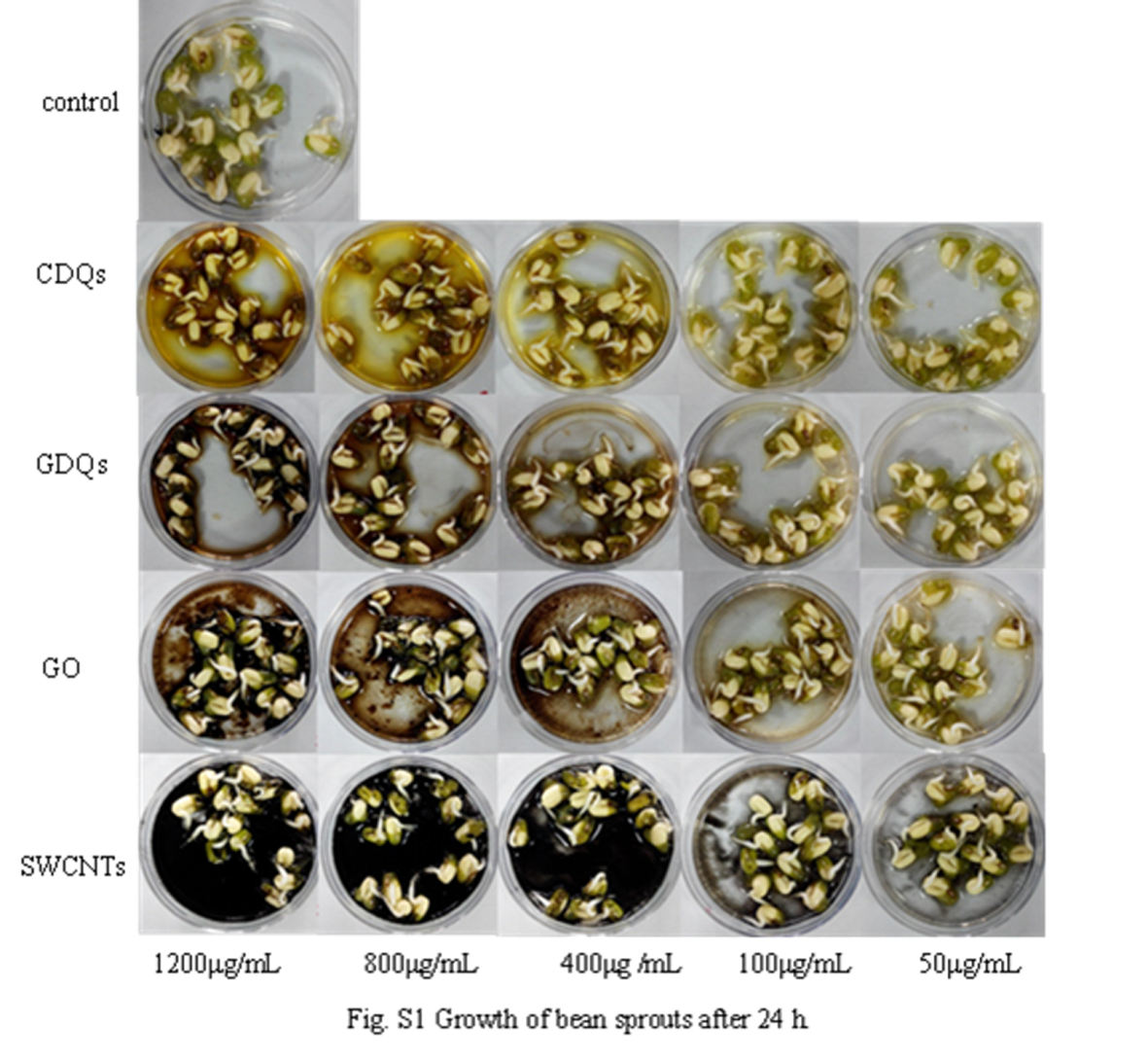


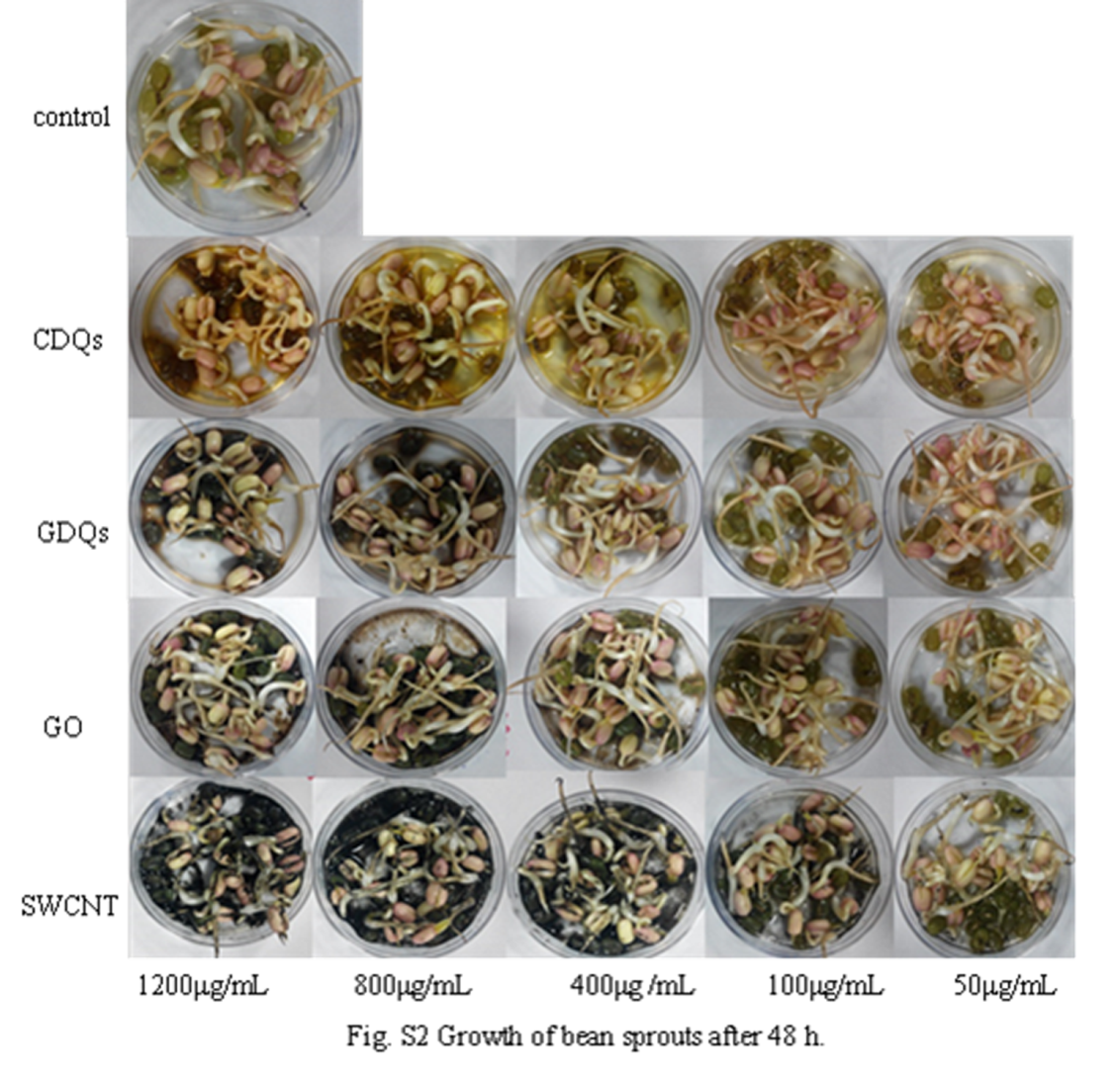


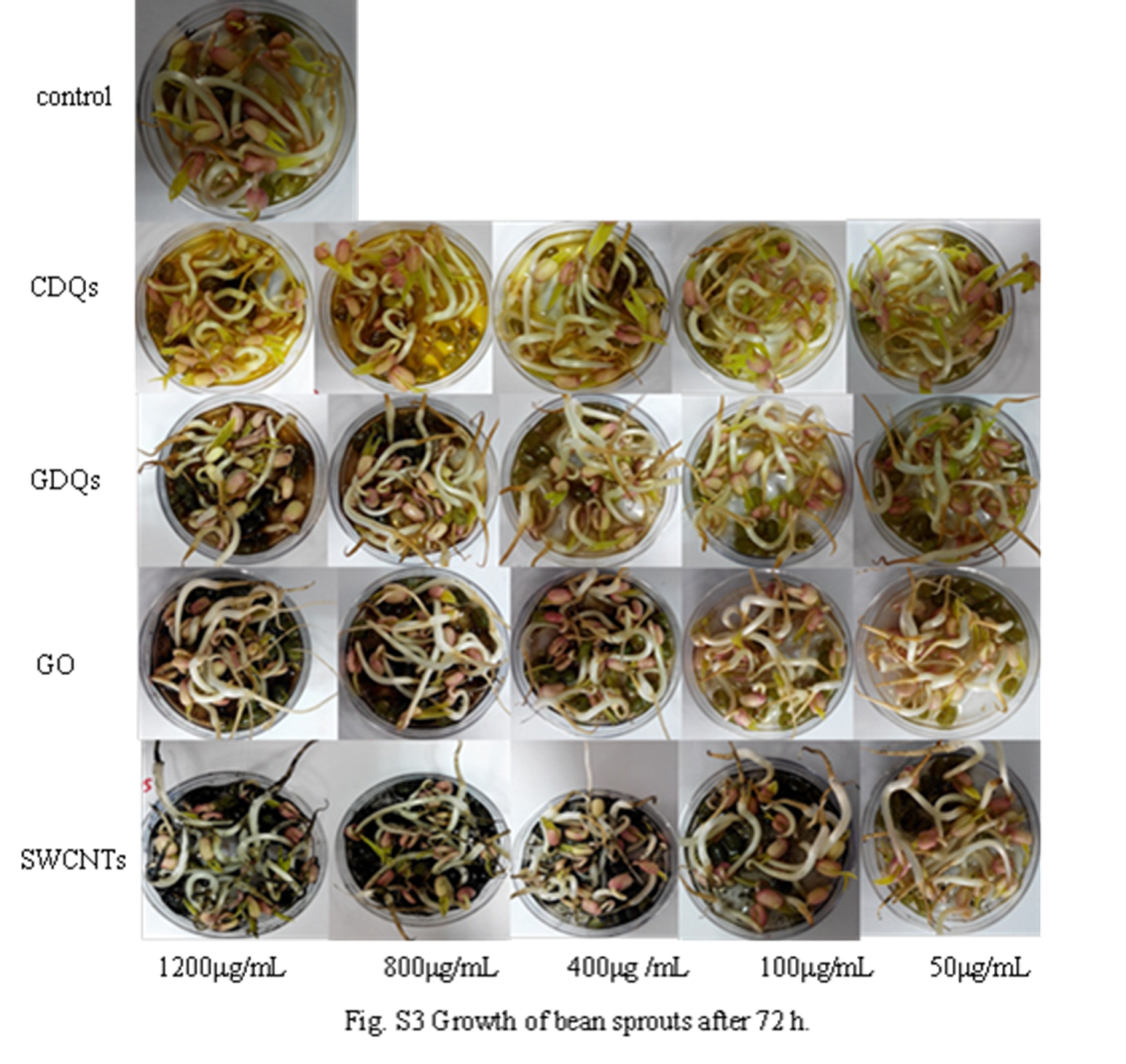


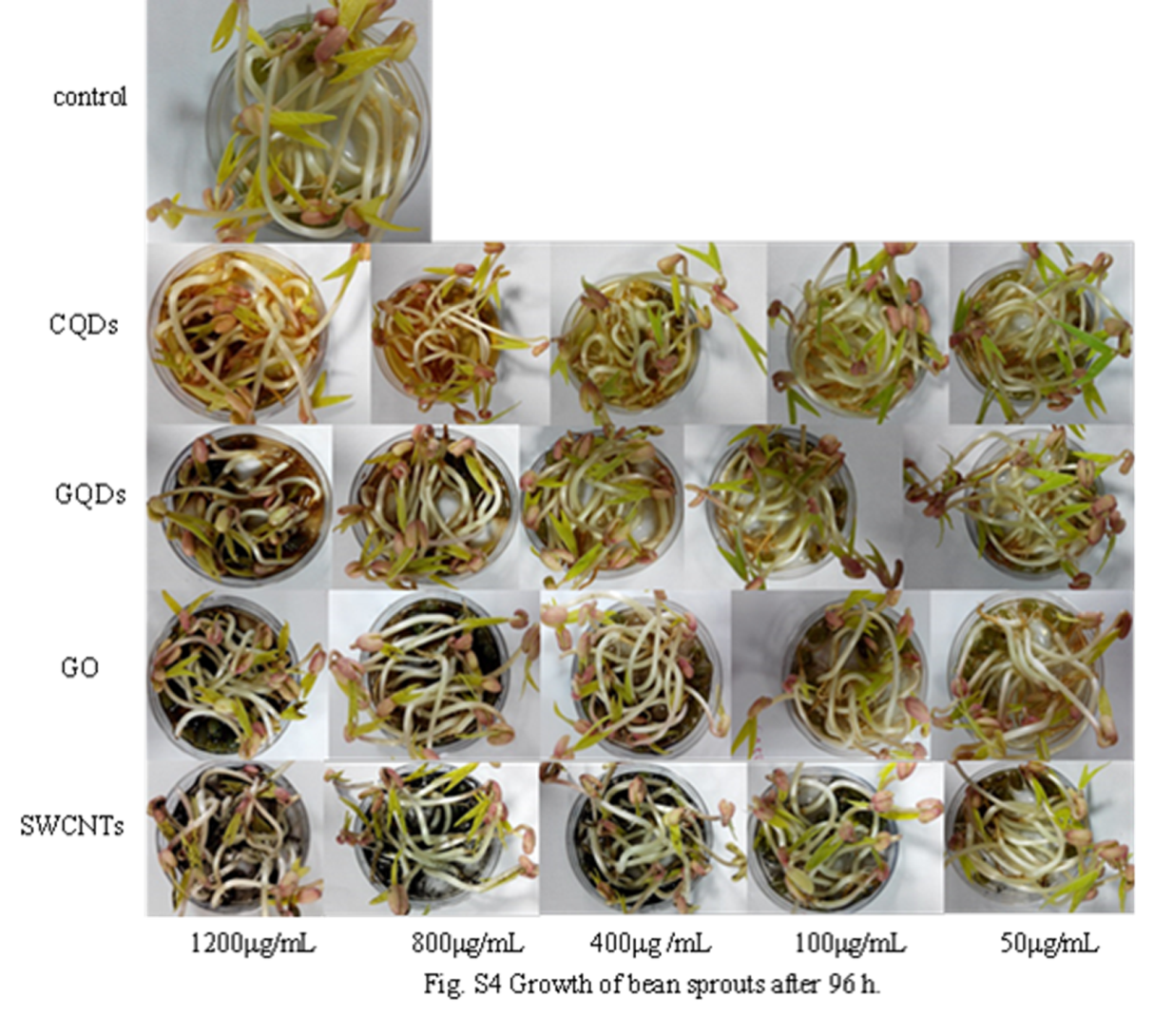

Supplement: Additional file 1: Figure S1-S4 — Photograph of the bean sprouts. Typical growth situations of the bean sprouts with time. [file 1556-276X-9-583-S1.docx]
